# Butler Hospital for the Insane

**Published:** 1847-04

**Authors:** 


18. *Butler Hospital for the Insane*, *Providence*, *R. I.*—We understand that this Institution will probably be open for the reception of patients in October next. We shall be much surprised if it does not take immmediate rank among the first in the country. Dr. Ray, is zealously engaged in completing it in the best manner. We purpose hereafter to notice more particularly the excellence of its arrangments, and the beauty of the surrounding scenery, etc.—*Ibid.*

